# Chemistry and Physiology

**Published:** 1858-11

**Authors:** 


CHIEFLY FRENCH, GERMAN, AND ITALIAN; OR, BI-MONTHLY NOVELTIES IN MEDICAL, SURGICAL, OBSTETRICAL, AND CHEMICAL SCIENCE. BY EMIL FISCHER, M.D.

### CHEMISTRY AND PHYSIOLOGY.

### I.—*Researches on the Chemical Composition of the Brain.* By M. Müller.

In his first memoir on the composition of the brain M. Müller states—1. That the human brain contains a small quantity of creatin; 2. That the creatin does not exist in the brain of the ox, where it is substituted by leucin, or a substance homologous to it; 3. That in the human brain, and in that of the ox, much lactic acid, and a small quantity of volatile fatty acids, $C^n H^n O^4$, is found; 4. Finally, that the brain of the ox contains a small quantity of uric acid, and a notable quantity of inosit, $C^{12} H^{12} O^{12}$.—(*Annales de Chemie et Physique;* and *Journ. de Pharm. et Chem.*, July, 1858.)

### II.—*On a New Basic Substance Extracted from Muscles.* By A. Strecker.

In the syrupy liquor of the extract of muscular tissue the author has found a new substance, feebly basic, which he calls *sarkin.* He extracted it in the form of a white crystalline powder, which resists a heat of 150° R., and is decomposed without melting at a higher temperature. It is soluble in 300 parts of cold water, and in 900 parts of alcohol when heated. Its formula is $C^{10}$, $H^4$, $Az^4$, $O^2$. In the flesh of the ox it is contained in the proportion of at least 0·22 to 1000. It has been found in the flesh of the ox and horse. In several particulars it resembles hypoxanthin, guanin, and xanthin.—(*Schmidt's Jahrbücher*, 1858, No. 1, p. 15; and *Journ. de Physiol.*, April, 1858.)

### III.—*Influence of the Spinal Marrow on the Brain.* By Dr. Brown-Séquard.

Dr. Brown-Séquard found that on section of one-half of the spinal marrow in the lumbar region, the skin of the face acquired, on the side on which the operation was performed, the power of causing epileptiform convulsions when irritated; this cutaneous zone seems to have undergone a great modification, which is attested by the great number of lice which prefer to meet on this spot. Sometimes the cornea ulcerates or becomes opaque; at other times, and always on the side of the section, convulsions supervene in the muscles of the face; they are of short duration, and are followed by an equally transitory augmentation of the secretion of nasal mucus. As all these disturbances show themselves on the same side with the section of the spinal marrow, a transmission through the sensitive nerves is out of question, for they cross each other in this structure; the modifying action passes through the brain to certain nerves of the face of the same side, following, probably, conducting elements which do not cross.—(*Journ. de Physiologie*, April, 1858; and *Archives Générales*, July, 1858.)

IV.—*Measurement of the Sensibility in the Morbid State.* By DR. BROWN-SÉQUARD.

The process employed by Dr. Brown-Séquard is the same used by M. Weber for the measurement of the normal sensibility. It is known that M. Weber has determined for different regions of the body, the distance beyond which two points of the compass applied on the skin produce a double impression. Dr. Brown-Séquard uses a graduated compass with pointed branches, an imitation of the æsthesiometer of Dr. Sieveking. He thinks that by this means we may be enabled—1. To recognize the existence of a diminution or augmentation of tactile sensibility in cases where it would be impossible to ascertain it by other means; 2. To find out exactly the degree; 3. To follow in a precise manner the increasing or decreasing course, and to judge of the action of medicines. In a case of hyperæsthesia of the foot, the patient, who suffered from paraplegia, was found to perceive the sensation of two points at a distance of only five millimetres in the dorsal region of the foot, while in the normal state the sensation of two points is only perceived when they are at least twenty-four to twenty-eight millimetres distant from each other.—(*Journal de Physiologie,* April, 1858; *Archives Générales,* August, 1858.)

---

### PATHOLOGY AND THERAPEUTICS.

V.—*Serous Sac of the Middle Ventricle of the Brain.* By PROF. FÖRSTER.

Prof. Förster reports the following very remarkable case: A chronic collection of serum in the ventricles had, by a peculiar distribution of pressure, distended the floor of the middle ventricle, and had pressed it outward, so as to form a pretty large sac of serum between the brain and its basis, which necessarily exerted considerable pressure upon the neighboring parts, particularly, however, upon the optic tracts and their chiasms. The patient, a man forty-four years old, of intemperate habits, complained first of a gradual decrease of his power of vision; at the same time headache set in, which was occasionally very violent, but afterwards abated. To these symptoms were added attacks of giddiness, and from time to time convulsions and vomiting. The pupil was dilated and rigid; the gait unsteady, with a prevalent debility of the right side of the body. From a seton applied to the neck, erysipelas was developed, which spread gradually over the head, chest, and left arm, and was accompanied by violent fever and delirium; after repeated convulsions and violent dyspnœa, death ensued. The sac mentioned above, bulged out between the two crura of the chiasms; was ten lines long, and eight lines in height and width; its wall was continuous at the base with the floor of the third ventricle. The microscopic investigation of the optic nerve revealed decrease of the nerve-fibres, and increase of the interstitial areolar tissue. The left half of the cerebellum was somewhat enlarged, and the medulla oblongata pushed thereby somewhat to the right side. In cutting through the midst of the lower part of the left small hemisphere, a circumscribed, but not encysted tumor, of the size of a walnut, was discovered near the periphery; it was found to be a tetangiectasis, consisting of an interlacement of prodigiously enlarged sinuate capillaries; by the confluence of such sinuses a body of the size of a pea, resembling a cyst, and containing fluid blood, was formed in the middle of the tumor. The vessels of the tumor continued at the periphery into the vessels of the cerebral substance. The cerebral tissue around it was softened.—(*Virchow's Archiv. für pathol. Anatomie und Physiologie*, 1858, xiii. 1.)

### VI.—*Anæsthesia Universalis Peripherica.* By DR. BINZ.

A servant girl, nineteen years of age, of good constitution, took cold, and was found by Dr. Binz in the following condition: Moderate fever, and the usual subjective symptoms; the whole skin quite insensible to pricks with a needle, with the exception of a few places in the region of the trigeminus, where some sensibility existed; this anæsthesia was most decided at the extremities; also the apertures of the mucous membranes were insensible; deep pressure upon the muscles was, however, felt; sense of hearing and sight in normal condition; taste and smell abolished; power of motion retained in every part of the body. Patient took magnes. sulphas, and tea of flor. sambuci. The anæsthesia remained the same for four days; after that it gradually disappeared in indefinite succession under aid of energetic friction, so that the extremities regained their sensibility last. In eight days the patient was cured.

Dr. Binz considers this anæsthesia of rheumatic origin, as neither hysteria nor deception could be assumed. A similar case was reported in the *India Journal*, January, 1836, where one-half of the body of the patient became also completely insensible in consequence of exposure to the moist and cold night air.—(*Deutsche Klinik*, 1858, xii., and *Schmidt's Jahrbücher*, 1858, 6.)

### VII.—*Pericarditis, with Acute Fatty Degeneration of the Heart.* By PROF. VIRCHOW.

A young man, twenty years of age, was attacked with pericarditis, in consequence of acute articular rheumatism, and died ten days after the attack. Prof. Virchow found the muscular structure of the heart degenerated in a manner never described before. The pericardium contained sixteen ccm. of a thin, blood-red liquid, in which, however, on microscopic examination, no blood-corpuscles could be detected, while the commencement of cadaverous changes was indicated by the presence of long vibriones, by the influence of ammonia, and perhaps by leucin being present only in small quantity. The surface of the pericardium was much roughened by thick layers of fibrin, which could be separated from the wall only by force. The muscular structure of the heart appeared pale, flaccid, and somewhat spotted, but on closer inspection it was found that the external layer of muscles underneath the whole extent of the pericardium had assumed a pale-yellow, muddy appearance. Microscopic examination showed that here all the primitive fasciculi had undergone so complete a fatty metamorphosis, that their inner structure was not any more perceptible. On penetrating to the deeper layers this metamorphosis gradually decreased; there was, however, no part of the muscular structure of the heart that had been quite free from fat-granules in the interior of the primitive fasciculi.

In the second case Prof. Virchow found, on autopsy, an unsuspected purulent pericarditis, in which the exterior stratum of muscles revealed the same change. In several other cases, on the contrary, partly of a less severe character, partly such in which the pericarditis was merely a complication of some other serious disease, this change was either quite absent or only little developed. The author is therefore of the opinion that this peculiar fatty degeneration, which penetrates from the periphery to the interior, is conditioned directly by the pericarditis, and that to it in particular the debility and paralysis of the muscles of the heart are to be attributed, which so much endanger the patient's life. It is, according to its seat, the opposite of the chronic fatty degeneration which accompanies the dilatation of the ventricles, and which is likewise attended by debility; the latter commences at the endocardium, usually in separate spots, a circumstance by which it is distinguished from the more uniform fatty degeneration of the inner muscular layers as observed in old people. The pericarditis is generally complicated with the disease of the inner structure of the heart, in those cases in which the action of that organ is subjected very early to an unusual acceleration. Just those cases of acute articular rheumatism offer the greatest danger in which the fever is very violent from the beginning. According to experience, that treatment has the best prospect of success which decreases the violence of the heart's action the quickest, and restores rest to the muscles most completely. Thus all the circumstances under consideration point out that the mechanical feature of the disease plays the principal part in regard to its etiology and prognosis; a quick and decided treatment is therefore of the greatest importance.—(*Virchow's Archiv. für pathologische Anatomie und Physiologie*, 1858, xiii. 2 and 3.)

VIII.—*Observations on Pulmonary Tuberculosis.* By PROF. LEBERT.

Prof. Lebert observed in his clinic, during the years 1855 and 1856, not less than thirteen cases of acute tuberculosis of the lungs, of which three occurred in male, and ten in female patients. He found, on experience, that the disease was capable of a cure. In persons who had died of other diseases he frequently met, on autopsy, with numerous miliary tubercles, which were shriveled and imbedded in pigmentary matter, without symptoms of tuberculosis having been manifest during the latter part of their lives. A women, thirty-six years of age, who had all the symptoms of acute pulmonary tuberculosis of typhoid character, began to recover after four weeks, and was dismissed cured after two months. In a case which terminated under typhoid symptoms, granulations were disseminated through the liver, spleen, and kidneys; tubercles were found on the great curvature of the stomach near the pyloric region, and granulations existed on the mucous membrane of the intestines. In chronic tuberculosis the author did not obtain the favorable results from the use of cod-liver oil which English physicians boast of; he recommends, however, its use as nutriment, in combination with bitters, tonics, ferruginous preparations, and, as a palliative, opium.—(*Virchow's Archiv.*, 1858, xiii. 2 and 3.)

IX.—1. *Acute Yellow Atrophy of the Liver with Icterus.* By Dr. Muhlig, of Constantinople.

2. *Similar Case.* By Prof. Bamberger, of Würzburg; *with an Analysis of the Blood, etc.* By Prof. Scherer.

Case I.—An English woman, twenty-one years of age, had suffered much of late from misery and grief. She had arrived at the last month of her first pregnancy, when her conjunctiva became icteric and her temper more irritable than usual. She was delivered without accident, and had no bad symptoms for three days; but in the night of the fourth she was seized with violent agitation and delirium. A physician was called in, and prescribed an emetic; afterwards the patient was put under mesmeric influence. Dr. Mühlig saw her on the fourth day. He found her lying on her back, the eyelids closed, the pupil dilated and immovable; the skin and conjunctiva colored light-yellow; expression and physiognomy apathetic; persistent trismus; from time to time hiccoughs, and slight flexing movements of the forearm. The patient was in profound coma; no sensibility betrayed on pressure in the hypochondriac regions. Involuntary evacuations; retention of urine; temperature of skin not sensibly augmented. Percussion gave a tympanitic sound in the epigastric, and an obtuse tympanitic in the hepatic region, over the inferior ribs, to an extent of about two fingers square; higher up dullness to the extent of about four centimetres. Purgative injections and sinapisms. Died the following night.

*Autopsy.*—Absence of cadaveric rigidity. Heart normal, containing some liquid blood and very friable clots. The liver was reduced to nearly two-thirds of its normal volume, and was concealed in the depth of the right hypochondrium; its peritoneal coat was wrinkled; its left lobe represented merely a small appendix to the right lobe. The diminution of volume was remarkable, particularly in the thickness of the organ, which seemed flattened, with the borders flabby and hanging down. Its tissue was flaccid and without resistance, and presented a uniform light-yellow color, except in some points where it was purplish-red, and gave out neither blood nor bile. Gall-bladder distended by a grayish, flaky liquid; biliary ducts contracted but permeable; spleen enlarged and softened; stomach and large intestines contained grayish mucus, mixed with a blackish liquid resembling coffee-grounds, but there were no hemorrhagic erosions present. On microscopic examination the hepatic cells were found completely destroyed; only a molecular, yellowish, uniform detritus, and some oil-globules were visible.—(*Gazette Médicale d'Orient*, March, 1858.)

Case II.—M. T., twenty-eight years old, had recovered some weeks ago from a pelvic abscess which had discharged through the vagina. She lived in trouble, and seemed to have been subjected to strong emotions in the first days of her new malady. On the 3d of October, 1856, she became icteric; she complained of cephalalgia, tinnitus aurium, prostration, and pain in the extremities. On the 6th the liver extended beyond the ribs, and its border could be easily felt on palpation. On the 9th it could not be reached except by thrusting the fingers deeply into the hypochondrium; also percussion proved a diminution of the volume of the liver. On the 15th evening delirium set in, then agitation, and on the 16th, the day on which the patient entered the hospital, she was in a comatose state. Lying on her back, she seemed to be unconscious of what was passing around her, and only answered in monosyllables, if questioned; sensibility was, however, retained; convulsive attacks from time to time; intense icterus of the skin; pulse sixty-eight, very small; temperature in the axilla, 35·5° R. Right hypochondrium not painful on pressure; percussion dull only on the external side of the hepatic region to the extent of seven centim., more internally somewhat tympanitic; to the left of the median line all dullness disappeared; the stools only little colored; urine voided involuntarily, and of icteric tint. (Ether; wine; injections of vinegar; sinapisms; cold affusions.) A little improved on the 17th. On the 18th icterus more intense; depression more profound; pulse seventy; temperature 36·2°. (Calomel; affusions.) Much agitation during the night. On the 19th grayish, involuntary stools; nearly absolute coma; hepatic dullness less extended than previously; pulse eighty-four; temperature 36°; hands cold, cyanotic; the patient has, nevertheless, some appetite. On the 20th the stools were quite colorless; agitation alternated with prostration. On the 21st nothing could awake the patient; the skin, if raised into a fold, would resume its place only slowly; a diphtheritic ulcer had formed on the interior side of the lower lip; sanguinolent sputa; pulse 120. Died the same evening.

*Autopsy.*—Icteric coloration of all the organs, with exception of the brain, which contained only little blood, but presented no other anomaly. The left heart and the vena cava contained liquid blood mixed with some soft clots. Underneath the endocardium of the left ventricle a great number of ecchymoses were observed; the spleen was voluminous; liver atrophied; the diminution of volume affected particularly its left lobe, and the thickness of the organ, which, at a point of the right lobe, did not exceed eight centimetres. Its tissue was remarkably flaccid and friable; its color orange-yellow, mixed with red in the left lobe. The lobules were nearly effaced, except toward the posterior border of the right lobe, which contained also more blood than the rest of the organ. The branches of the portal vein were empty, otherwise healthy; the hepatic ducts collapsed, and for the most part empty. The gall-bladder contained only half a teaspoonful of thick bile of greenish-gray color; hepatic artery permeable. The microscopic examination showed that the lobules of the liver were preserved nearly everywhere. The cells, which occupied their centre, were much colored by bile, while those at the periphery were, for the greater part, destroyed, and replaced by fatty and pigmentary granules. The contents of the large intestines were quite colorless, except in the region of the ileo-cæcal valve, where they were slightly colored by bile. The chemical analysis of the different liquids and solids of the economy, by Prof. Scherer, was made particularly to search for leucin and tyrosin. It was found that—1. The urine contained leucin, and biliary pigment; neither albumen, nor tyrosin, nor tauro- and glycocholic acid. 2. The blood: leucin more abundant in the venous blood than in that of the left heart; no tyrosin nor hypoxanthin. 3. The bile: taurocholic and glycocholic acid; neither tyrosin nor leucin. 4. The spleen: hypoxanthin and leucin, little tyrosin, traces of oxalate of lime. 5. The liver: much leucin, and tyrosin, and hypoxanthin.

Professor Bamberger remarks, in regard to the etiology of acute atrophy of the liver, that the patient was debilitated by a previous disease, and had been under the influence of strong emotions. These circumstances are the only ones which had been pointed out as the cause of this disease, and they are, in fact, very usual. In the patient of Dr. Mülig they have been likewise noticed. Dr. Bühl has counted the acute atrophy of the liver under the general diseases.—(*Zeitschr. f. Rationelle Med.* New series, viii.) It is, according to this author, only one of the elements of a general atrophy of rapid course. This hypothesis, based as it is upon an isolated case, in which, besides the atrophy of the liver, atrophy of the brain and fatty degeneration of the heart was found, is in opposition to numerous facts in which the liver alone was found atrophied, and, in these cases, it is at least useless to assume a general primitive disease as cause of this atrophy. The intense yellow color of the hepatic cells yet preserved seems to idicate that the liver continues to secrete bile, at least for some time, and that some obstacle opposes the discharge of this liquid. The granulations of biliary pigment which are met here and there, speak likewise in favor of this hypothesis. But this obstacle can have its seat evidently only on a level with the origins of the biliary ducts, because in the rest of their course they are found permeable. Now, in the second case, the destruction of cells existed particularly on a level with the periphery of the lobules, that is to say, in the points where the biliary ducts originate, according to Kölliker, without communicating directly with the cells of the centre. It can thus be assumed that these ducts are obliterated by the molecular detritus of the destroyed cells, or that the bile secreted in the middle of the lobules cannot be any more conducted to their periphery by means of imbibition. The cause of the destruction of the cells Professor Bamberger considers to be an acute parenchymatous inflammation; a process probably takes place analogous to that observed in Bright's disease, in which the inflammation is likewise accompanied by a fatty infiltration, and then, by the destruction of the cells, having charge of the secretion.—(*Verhandlungen der physikalisch-medizinischen Gesellschaft in Würzburg.* 8vo., pp. 268 and 281; *Gazette Hebdom.* of July, 1858.)

X.—*On Nutmeg Liver.* By DR. ANT. REZZONICO, of Milan.

After a short review of the more recent views on the structure and functions of the liver, as well as on the so-called nutmeg liver, the author dwells upon the changes in the circulatory and respiratory systems accompanying the last-mentioned condition of the liver. Dr. Rezzonico has made the autopsy of forty patients who died of diseases of the heart and lungs, and arrived at the following results:—The nutmeg liver is found nearly constantly in tuberculosis, (among twenty-four tuberculous subjects, nineteen times nutmeg liver, three times fatty liver, and twice tuberculosis of the liver;) on the contrary, rarely in diseases of the heart, (twice in eight cases;) in patients of the latter kind, a congested or hypertrophic condition of the liver is more frequent. Nutmeg liver was found in three cases of emphysema, and not at all in acute diseases of the lungs. Neither with fatty nor nutmeg liver did the patients complain during life of symptoms from this organ. Dr. Rezzonico believes that the myristicatio hepatis is only a physiological condition, a consequence of excessive action the liver has to take upon itself, in order to compensate for the diminished functions of the diseased lungs. The greater amount of blood carried to the organ in consequence of it, brings about a more active nutrition, and an enlargement of the secreting gall-ducts. The fact that in infants nutmeg liver is not observed, in spite of the increased activity of the organ, appears not to be contradictory to this view; it follows from the chronic course of the causal diseases, that a longer space of time is necessary to develope myristicatio hepatis. In infants the increased activity of the liver lasts only a short time, after which heart and lungs resume their normal functional state; the liver obtains thus a period of rest, during which a diminution of its volume takes place.—(*Ann. Univers.*, Gennajo, 1858.)

XI.—*Observations on Cholera.* By PROF. LEBERT.

Prof. Lebert has laid down his observations, made during the late cholera epidemic in Zürich, in the following report:—In one-third of the cases, the usual antecedent diarrhœa was absent, and equally so in patients who recovered as in those who died. If it was present, its duration was generally between one and three days; in nearly one-fourth of the cases, however, between half a week to a whole one. The temperature never fell as much as it appeared, generally not underneath 27·2° R., only once to 24·8° R. In one case the face and extremities appeared warmer to the touch of the hand than immediately before death. Although the suppression of urine was generally complete in bad cases, Prof. Lebert has, nevertheless, observed a fatal issue in cases in which urine was voided. In six patients who afterwards recovered, he found the urine merely reduced in quantity, and containing albumen. The secretion of urine commonly appeared again in the course of the third or fourth day, in only one case after four, and in another after six days. The typhoid course of the disease seems to be owing more to the entire pathological process, than especially to uræmic intoxication. In reference to *pathological anatomy*, the rarity of diphtheritic processes is to be remarked; only once, croup of the larynx and œsophagus occurred, and in only one patient recent tuberculosis set in. Among the more frequent anatomical changes found in cases of rapidly fatal termination, Prof. Lebert points out particularly the injection of the peritoneal surface of the small intestines, which was often covered with an adhesive secretion. After an early death, Brunner's duodenal glands were always found to be swollen. The isolated and agminated glands had a muddy, milky appearance, and were tumefied, but did not show real signs of inflammation, and could be injected with perfect ease; they were apparently œdematous, and the molecular nucleoli and nuclei seemed to be augmented. A decrease in their tumefied condition took place sometimes as early as from twenty-four to forty-eight hours, in other cases not after three days; they became then more flat, somewhat corrugated, some time afterwards firm, granular, still prominent, but diminished in circumference; their color was then more yellowish-gray, and assumed, some time later, in many cases a slaty tint; in other cases, a grayish-black, brown, or reddish-brown pigment was found on these glands, particularly if ecchymoses had formerly existed; in the second week the tumefaction usually disappeared, and an increased density and abnormal pigmentation only remained. In the first period, the patches of agminated glands showed frequently a net-like appearance. Also in the large intestines, the glands were swollen, prominent, lenticular, or having a blackish or reddish aperture; at a later period they decreased in size, and revealed retrogressive changes similar to the glands of the small intestines. Already, sixteen to twenty-four hours after the commencement of the disease, the volume of the kidneys had increased; they were in a state of general hyperæmia, with ecchymoses here and there; as early as the second half of the first day, Prof. Lebert found the cortical substance discolored, but otherwise merely increase of the epithelial cells, with albuminous granules; only in rare cases exudation cylinders in the uriniferous tubes. In the course of the second day these cylinders increased in quantity; on pressure, a turbid albuminous urine containing cylinders, and sometimes crystals of uric acid, was discharged from the papillæ; the mucous membrane of the pelvis of the kidney and of the calices was usually very hyperæmic, covered with numerous minute ramifications of vessels; in the course of the third day the discoloration was more constant, more general, and well marked; the cortical substance was of a pale, yellowish color, and granulations were seen in it here and there; the hyperæmia was unequally distributed; the surface became unequal and rough; about this time the secretion of urine generally reappeared, being charged with albumen, cylinders, epithelial cells, and crystals of uric acid; the exuberant growth of cells, the desquamation of epithelium, and the formation of exudation cylinders had reached a certain intensity. Only in the course of the third day, and toward its end, the fatty degeneration commenced. In the typhoid state, and generally in incomplete convalescence, these changes rapidly increased; the kidneys were larger than in normal condition; the vascular islets became rarer and rarer; the granulations more and more numerous; the substance of the kidney became softer, was more easily torn, and infiltrated with a turbid, yellowish liquid containing numerous elements of fat. On *chemical investigation*, the liver of a patient who had died some time after the attack, yielded an alcoholic extract which possessed a peculiar urinous odor, and contained much leucin; the presence of tyrosin and urea could not be satisfactorily proved. In another case in which the disease had terminated fatally within eight days under the appearance of typhoid symptoms, the liver, spleen, and kidneys were examined. The extract of the liver had not the urinous odor, and only after having stood for some time, a globule of leucin was here and there detected; tyrosin was not present; uric acid only in small quantities. The spleen contained leucin, inosit, uric acid, and biliary pigment; the kidneys a great portion of urea, some leucin, biliary pigment, uric acid, no inosit. In regard to *treatment*, Prof. Lebert recommends to pay particular attention to the diarrhœa from the beginning; in many cases Argent. Nitr., gr. ss to grs. iij daily, in the form of pills, was sufficiently efficacious, and was used in case of very exhausting evacuations, in small clysters containing grs. iij—iv of the salt. Also opium was used in different forms, partly alone, partly combined with tannic acid and Argent. Nitr. During the attack itself, ice, and liquids containing carbonic acid, brought the greatest relief; large sinapisms were applied to the epigastric regions and the extremities. Clysters of Argent. Nitr. and opium appeared to be of use only after the great frequency of the evacuations was somewhat diminished. Toward the end of the attack, small doses of opium in Infus. flor. Tiliæ were sometimes very serviceable. For the decrease of temperature, external heat and tea of lime-blossoms, with fifteen drops of Liq. Ammon. anisat. for each cup, were ordered. During the existence of typhoid symptoms, the nausea and anorexia were allayed by Seidlitz powder, ice, carbonic acid water, and later, by bitter and aromatic infusions. Gentle cathartics, particularly pills of rhubarb and aloes, were here of use. Enemata containing ether (Aether. Sulphur, ʒj–ij to ice water, ʒiv,) were applied with great success in very troublesome tympanites with abdominal pain. Inflammatory affections of the chest were treated with cups and Inf. rad. Ipecac.—(*Virchow's Archiv. für Patholog. Anatomie*, &c., 1858, xiii. 2 and 3.)

XII.—*On Prolonged Constipation.* Reported from the Clinic of PROF. TEISSIER, by DR. COUTAGNE.

Throwing the constipation resulting from organic lesions of the intestines out of consideration, Dr. Teissier establishes the following varieties of constipation, which are founded on the causes they are produced by. Constipation can thus depend—1. Upon alteration of the mucous secretion, such as diminution of the intestinal exhalation, or modification in the composition of the mucus, etc.; 2. Upon functional disorders of the liver, which does not pour out a sufficient quantity of bile into the intestines; 3. On inertia of the intestinal contractility; 4. On a spasmodic state of the muscular coat of the intestines. The most frequent cause of constipation is the indolence and inertia of the muscular fibres of the intestine. This variety is frequently combined with those which result from derangement in the digestive secretions, and is observed ordinarily in old people, in persons of sedentary habit, and those who repose too much in bed. After a review of the well-known symptoms of constipation, Dr. Teissier considers the treatment of the disease, which he bases upon the etiological distinctions established above. For constipation, in consequence of alteration of the mucous secretion, he recommends emollient injections with honey or oil, and light laxatives. For constipation from derangement of the biliary secretions, drastics, rhubarb, aloes, calomel, extract of ox-gall, etc. In habitual constipation dependant upon indolence of the intestine, Dr. Teissier objects to the use of purgatives, and even to warm emollient enemata or laxatives, as they only augment the evil; purgatives excite the intestinal secretion only momentarily to diminish, and even suspend it afterwards, while warm and emollient injections, although bringing temporary relief, tend to weaken the intestinal tunics, and to put them in a state of atony. For patients of this kind M. Teissier recommends—1. To regulate the habit of the intestinal functions by going to stool each day at a fixed hour, and making prolonged efforts to provoke contractions of the large intestines, as recommended by M. Trousseau; 2. Injections of cold water; 3. Nux vomica in very small doses each morning; 4. The *tea of Saint-Germain*, which Prof. Teissier considers one of the most efficacious remedies, having used it for ten years with great success. The original formula, which we copy from Hufeland's "Enchiridion Medicum," is the following:—

℞.—Folior. senn. ℥iv;
Diger. cum spirit. vin. rectif. per
Horas xxiv. Post digestionem exsicca
Sine calore. Adde
Flor. sambuc. ℥ijss;
Semin. fœnicul.
——— anisi āā ℥j;
Cremor. tartari ʒvi.

S. An infusion to be made with five cups of boiling water on two ounces of these ingredients. Digest for ten hours with gentle heat without boiling, and then decant the clear liquid, of which half a cup is to be taken every morning. 5. Coffee with milk; 6. Bran-bread; 7. White mustard; 8. Ervalenta. For the treatment of constipation dependant upon nervous erithism of the intestines, a form frequently met with in hysteric, neuropathic females, the best remedy is belladonna in fractional doses: one centigramme of the extract every day.—(*Gazette Medic. de Lyon*, and *Gazette Hebdom.*, 25th June, 1858.)

XIII.—*On the Observations of Temperature in Patients.* By PROF. C. A. WUNDERLICH and DR. L. MEYER.

Already during the last century several eminent physicians endeavored to ascertain the temperature of the body in different diseases. These efforts, however, were soon abandoned again, and only quite recently the thermometric observations in patients have again received that attention which they so much deserve. Prof. Wunderlich has, in this respect, a rich experience at his command; accurate thermometric observations were regularly made in his clinic in more than 5000 patients during the whole course of their sickness, and also in private practice he has convinced himself of the practicability and usefulness of this means of investigation. He considers himself justified, therefore, to pronounce the view so generally taken, and recently advanced again by Lasègne, (*Arch. Génér.*, May, 1856,) that thermometric investigations would never become very important to pathology, as perfectly erroneous. It is true, that for the theory of diseases these observations of temperature have remained as yet without direct use, and that they do not throw much light at present upon the nature of fever, inflammation, etc., but also in reference to theory they have afforded facts which are of great consequence for many important questions in relation to pathological physiology. Of far greater importance, however, are they in a *practical* point; Prof. Wunderlich considers them even of more value than most of the other means of investigation, provided that also in local diseases the part taken by the whole organism is considered of sufficient moment. As proof of his statements, the author gives the following facts:—

1. The observation of temperature offers the most reliable means for deciding *the importance of a disease of recent origin;* with normal, or but little elevated temperature, the disturbances of health are, with some exceptions, (cholera, apoplexy, pulmonary hemorrhage, strangulated hernia, poisoning, etc.,) first of all of no importance; an elevation of 2° R. or more, announces, however, with certainty, the commencement of a serious disorder. This circumstance is a valuable guide, particularly in cases of children, in which, as is well known, an insignificant disease is frequently accompanied by violent symptoms, as also in reference to the continuation of the patient's business, to his departure, or transport, etc.

2. The observation of temperature points out frequently *important, though still latent disturbances;* an indisposition with much elevated temperature deserves always particular attention; in the state of reconvalescence from serious diseases a relapse, or a secondary disease, is frequently indicated first by an elevation of temperature. This is particularly the case in typhus; but also in intermittent fever an elevation of temperature without any other symptoms is frequently observed after an apparent cure, and a relapse can then only be prevented by continuing the use of quinine.

3. If the disease is developed the observation of temperature offers *the most reliable indications for the diagnosis.* Diseases in which the diagnosis of particular forms of the same or other of pathological processes can be decided in this manner are, according to Prof. Wunderlich, the following:—typhus, (exanthematic; enteric;) intermittent fever; pneumonia; meningitis, (at the base; at the convexity;) serous and purulent effusions in the pleura or pericardium; acute exanthemata; internal suppurations; peritonitis, (in lying-in women.)

4. The diagnosis being decided, thermometrical observations are of the geatest use in reference to the *prognosis. Intensity* and *character* of the disease, *its stage* the commencement of a complication which is often not indicated by any symptom, the usual *aggravation* and *increase* of the malady, as well as its *decrease,* can be recognized, as the author proves by many examples, the earliest and surest, sometimes even solely by the behavior of the temperature of the body. A certain height of temperature (about 34° R.) indicates, with certainty, a fatal issue; perseveringly high temperature (over 32·5°) makes the prognosis always more serious; a falling of temperature in a proper manner, however, permits predicting a favorable turn of the disease. In the state of *convalescence* changes of the temperature of the body deserve no less consideration as a means by which to recognize deviations from health, otherwise hardly perceptible. Insufficient falling of temperature indicates, in spite of apparent convalescence, an incomplete cure, and gives reason for fearing the development of a chronic disorder; even a small increase of temperature challenges precaution in regard to diet and regimen of the convalescent.

5. Another great advantage derived from thermometric observations is the proof of *a regular typic course of numerous febrile diseases;* it is true that physicians of a former period supposed it to exist, but it cannot be demonstrated with certainty but by accurate observations of the changes of temperature of the body. In the same way *deviations* from this regular typic course are best recognized by the use of the thermometer, and we are thus enabled to avert them by removing the causes, to neutralize their consequences, or also, as many of such irregularities are of a more favorable character than the normal course of the disease, to bring them on by therapeutical means.

6. From what has been said, the importance of thermometric observations for *therapeutics* is evident enough; they indicate where energetic interference is necessary, and when the disease may be left to itself again. The thermometer gives us, however, also reliable and accurate information in regard to the efficacy of certain remedies and methods of treatment used; for instance, on the effect of general bleeding, of calomel, digitalis, camphor, an emetic, and other energetic means in febrile diseases. The observations of temperature have, as the author shows, so great a value for therapeutics, particularly for the reason that the indications for treatment have more frequently to be derived from the general condition of the patient than from so-called local disturbances which usually disappear spontaneously, and in which direct interference is not of much benefit. —(*Arch f. Physiol. Heilk.*, N. S. 1, p. 5.)

Dr. Meyer considers observations of temperature in insane patients of very great use, as they aid the physician in determining whether there exists a direct disease of the brain, or whether the latter is affected merely by reflex action from another organ. In the former case a corresponding change of temperature is observed; if the delirium is accompanied by elevation of temperature without remission, a direct irritation of the brain exists. In reflex alienations, however, this change of temperature does not take place; if the temparature rises in these cases, it indicates the occurrence of a complicating disease. These statements, the importance of which for the prognosis and therapeutics of mental diseases is very evident, the author proves by a condensed report of numerous cases, (mania, progressive paralysis.) As the peculiar character of insane patients does not permit a long continuance of the usual mode of observation, viz., by placing the thermometer in the axilla, he prefers to insert it into the rectum.—(*Deutsche Klinik.*, 13, 1858; *Schmidt's Jahrbücher*, 6, 1858.)

